# Designing of an Efficient Whole-Cell Biocatalyst System for Converting L-Lysine Into Cis-3-Hydroxypipecolic Acid

**DOI:** 10.3389/fmicb.2022.945184

**Published:** 2022-06-27

**Authors:** Shewei Hu, Yangyang Li, Alei Zhang, Hui Li, Kequan Chen, Pingkai Ouyang

**Affiliations:** State Key Laboratory of Materials-Oriented Chemical Engineering, College of Biotechnology and Pharmaceutical Engineering, Nanjing Tech University, Nanjing, China

**Keywords:** ribosome binding sites, tricarboxylic acid cycle, reconstitute, α-ketoglutarate, whole-cell catalysis

## Abstract

Cis-3-hydroxypipecolic acid (cis-3-HyPip), a key structural component of tetrapeptide antibiotic GE81112, which has attracted substantial attention for its broad antimicrobial properties and unique ability to inhibit bacterial translation initiation. In this study, a combined strategy to increase the productivity of cis-3-HyPip was investigated. First, combinatorial optimization of the ribosomal binding site (RBS) sequence was performed to tune the gene expression translation rates of the pathway enzymes. Next, in order to reduce the addition of the co-substrate α-ketoglutarate (2-OG), the major engineering strategy was to reconstitute the tricarboxylic acid (TCA) cycle of *Escherichia coli* to force the metabolic flux to go through GetF catalyzed reaction for 2-OG to succinate conversion, a series of engineered strains were constructed by the deletion of the relevant genes. In addition, the metabolic flux (*gltA* and *icd*) was improved and glucose concentrations were optimized to enhance the supply and catalytic efficiency of continuous 2-OG supply powered by glucose. Finally, under optimal conditions, the cis-3-HyPip titer of the best strain catalysis reached 33 mM, which was remarkably higher than previously reported.

## Introduction

**SCHEME 1:**
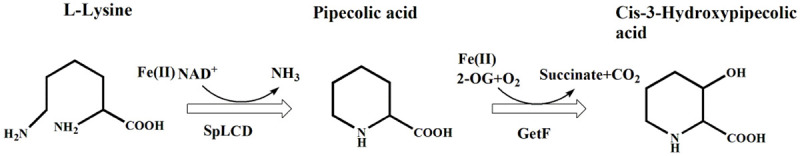
Biotransformation pathway from L-lysine to cis-3-HyPip by the whole-cell biocatalytic system.

Hydroxypipecolic acids (HyPips) are the core structure of many alkaloids and drugs, such as tetrazomine (Qi et al., 2021), non-ribosomal peptide GE81112 (Zwick et al., 2021), palinavir (Hibi et al., 2016), and relebactam (Miller et al., 2014). Currently, HyPips are predominantly produced using chemical methods, which have several disadvantages, including expensive substrates, complicated steps, negative environmental impacts, and challenges surrounding regio- and stereo-selectivity reactions that are inevitable during this process (Ahuja and Sudalai, 2015; Chavan et al., 2015; Zhang and Sun, 2020). Bioproduction of HyPips *via* microbial fermentation and enzymatic catalysis is an attractive alternative to chemical production methods, owing to its simplicity, stereoselectivity, mild reaction conditions and eco-friendly properties. L-pipecolic acid (L-Pip) is often used to produce HyPips through hydroxylation with Fe(II)/α-ketoglutarate (2-OG)-based oxygenase (Mori et al., 1997; Shibasaki et al., 1999; Hara and Kino, 2009; Mattay and Huettel, 2017; Lu et al., 2020). However, the high cost of substrate L-Pip restricts its practical application. To reduce the cost, we previously constructed a microbial cell factory that can convert economic L-lysine into cis-3-hydroxypipecolic acid (cis-3-HyPip) with whole-cell cascade catalysis (Hu et al., 2022; Scheme 1). First, L-lysine was converted to L-Pip through the cyclization deamination by lysine cyclodeaminase (SpLCD), which was further hydroxylated by Fe(II)/2-OG-based oxygenase GetF to form cis-3-HyPip. Although we used plasmids with different copy numbers to regulate the expression of SpLCD and GetF and systematically optimized the reaction parameters, the central problem in the co-expression system was always the imbalance caused by differences in a specific activity or expression level. The lowest reaction rate limits the overall reaction rate, resulting in L-Pip accumulation. Therefore, developing a tunable multi-enzyme coordinate expression system in *Escherichia coli* for cis-3-HyPip production remains a top priority.

Precise control of the expression levels of multiple genes is critical in co-expression biocatalysis (Chen et al., 2017), and one strategy is to use different types of vectors or co-express multiple genes through one or more vectors to avoid low expression of rate-limiting enzymes and overexpression of non-rate-limiting enzymes (Liu et al., 2019a; Yang et al., 2021; Hu et al., 2022). Other strategies are generally possible to precisely control certain enzyme expression levels by using different promoters and ribosomal binding sites (RBSs) (Jiang and Fang, 2016; Zhang et al., 2019). For example, researchers have found that using the RBS-optimized strain, the conversion rate of L-aspartate biotransformed from maleate was nearly 100%, with no intermediates or byproducts (Liu et al., 2019b). To further avoid pyruvate overoxidation, the RBS sequences of UtCAT with various translation initiation rates were designed using a recently developed biophysical model of translation initiation, yielding 59.9 g/L pyruvate (Li et al., 2020).

Moreover, GetF from *Streptomyces* sp. L-49973 (Binz et al., 2010) was a Fe(II)/2-OG-based oxygenase and requires the co-substrate 2-OG to undergo oxidative decarboxylation and form succinate, which is another limiting factor. An adequate supply of 2-OG derived from inexpensive substrates is necessary for a whole-cell catalysis system. Previously, researchers have used L-glutamic acid oxidase to catalyze the production of 2-OG from glutamate, requiring additional overexpression of catalase to eliminate the effect of hydrogen peroxide toward enzymatic activity (Sun et al., 2019). Increased gene expression increases whole-cell pressure, which may affect the catalytic activity. Normally, 2-OG is metabolized *via* the tricarboxylic acid (TCA) cycle. Thus, little flux typically enters the synthesis pathway of the desired product. To address this problem, a smart strategy was developed by constructing a modified TCA cycle that changed the role of 2-OG from co-substrate to cofactor and that regenerated 2-OG (Lin et al., 2015). For example, *E. coli* cells expressing deacetoxycephalosporin-C synthase (DAOCS) were developed as a whole-cell biocatalyst to convert penicillin G to G-7-aminodeacetoxycephalosporanic acid, where the TCA cycle was engineered *in vivo* by blocking the normal TCA reaction from 2-OG to succinate, effectively coupling it with the DAOCS-catalyzed reaction to form a modified TCA cycle (Lin et al., 2015).

In this study, a combined strategy to increase the productivity of cis-3-HyPip was investigated. Firstly, GetF and SpLCD expression was fine-tuned *via* RBS engineering to improve cis-3-HyPip production. Subsequently, an efficient and sustainable 2-OG supply system was constructed, and the TCA cycle was disrupted at the 2-OG to the succinate step, forcing the cycle to incorporate a GetF catalyzed reaction. We also overexpressed *gltA* and *icd* to strengthen the artificial TCA cycle and increase the yield of cis-3-HyPip.

## Materials and Methods

### Strains and Materials

The strains used in this study were listed in Supplementary Table 1, *E. coli* DH5α served as the host for recombinant DNA manipulation and plasmid construction. For cis-3-HyPip production, *E. coli* BL21(DE3) and derived strains were used. The plasmid pEcgRNA carrying the *ccdB* gene was constructed and maintained in *E. coli* DB3.1. The DNA polymerases used for polymerase chain reaction (PCR) including 2 × Phanta Max Master Mix, 2 × Taq Master Mix and ClonExpress One Step Cloning Kit were purchased from Vazyme (Nanjing, China). Molecular biological reagents, such as T4 DNA ligase and DNA gel extraction kit, were obtained from TaKaRa (Dalian, China). Isopropyl β-D-1-thiogalactopyranoside (IPTG), ampicillin, spectinomycin, and kanamycin were provided from Sangon Biotech (Shanghai, China). All other chemicals were purchased from Sigma-Aldrich (Shanghai, China) or Sangon Biotech and were of analytical grade (Shanghai, China).

### Media and Culture Conditions

All strains were grown at 37°C and shaken at 220 rpm in lysogeny broth (LB). Based on plasmid maintenance requirements, ampicillin (100 μg/mL), kanamycin (50 μg/mL), or spectinomycin (50 μg/mL) were added to the medium. For recombinant strain expression, overnight cultures were inoculated into medium [12 g/L tryptone, 24 g/L yeast extract, 0.25% (w/v) glycerol, 2.3 g/L KH_2_PO_4_, and 12 g/L K_2_HPO_4_, 2.0 mM MgSO_4_, and trace metal mix containing 0.05 mM FeCl_3_, 0.02 mM CaCl_2_, 0.01 mM MnCl_2_, 0.01 mM ZnSO_4_, and 2 mM each of CoCl_2_, NiCl_2_, Na_2_MoO_4_, Na_2_SeO_3_, and H_3_BO_3]_. Cultures were shaken at 200 rpm and incubated at 37°C until the OD_600_ reached 0.4–0.6. Cells were then induced with 0.3 mM IPTG for 24 h at 20°C. After the end of the cultivation, cells were harvested by centrifugation and washed twice with 0.85% NaCl solution.

### Gene Cloning and Plasmid Construction

The strains and plasmids used in the present study are described in Supplementary Table 1. The primers used for gene cloning in this study were synthesized at General Biol (Chuzhou, China). Primer sequences are listed in Supplementary Tables 2–6. The sequences of all constructed plasmids were verified using DNA sequencing.

#### Construction of Plasmids With Different Ribosomal Binding Site Sequences

Ribosomal binding site sequences with different initial translation rates were used, and the sequences were derived from the International Genetically Engineered Machine Competition (iGEM^1^). The template was the pETDuet-RBS-*getf*-RBS-*splcd* plasmid, and the substitution was done by PCR of the whole plasmid with a forward primer (X-F) and a reverse primer (X-R). All successfully constructed plasmids were transformed into *E. coli* BL21(DE3) to express the genes.

#### Construction of pEcgRNA Plasmids and Donor DNA

According to a previously described method (Li et al., 2021), the pEcgRNA-Δ*sucA* and pEcgRNA-Δ*aceA* were constructed using the pEcgRNA plasmid. Positive clones were selected on LB plates supplemented with 40 μg/mL spectinomycin. Two homologous arms and the sequence to be inserted were separately amplified and then fused together by overlapping PCR to create donor DNA for integration. The PCR products were purified by gel extraction prior to electroporation.

#### Construction of a Rate-Limiting Enzyme Plasmid in Tricarboxylic Acid Cycle

The citrate synthase gene (*gltA*) was amplified from the genome of *E. coli* BL21(DE3), digested with *Eco*RI and *Hin*dIII, and ligated to the plasmid pCDFDuet-1-*gltA*. The isocitrate dehydrogenase gene (*icd*) fragment was digested with *Bgl*II and *Xho*I and inserted into the pCDFDuet-1-*gltA* digested with the same enzymes, resulting in the vector pCDFDuet-1-*gltA*-*icd*.

### Production of Cis-3-Hydroxypipecolic Acid by Using Different Ribosomal Binding Site Sequence Plasmids

The recombinant plasmids were introduced to *E. coli* BL21 (DE3) and then the cells were induced with 0.3 mM IPTG for 24 h at 20°C and collected by centrifugation. Under the same reaction conditions, the ability of recombinant strains was evaluated in terms of product yield. The reaction system consisted of 50 mM L-lysine, 60 mM 2-OG, 10 mM Fe^2+^, 10 mM L-ascorbate (VC), 0.1% (wt/v) trition and cells with a final OD_600_ of 30 in PBS buffer (50 mM). The reaction was carried out in a 50 mL Erlenmeyer flask for 20 h at 30°C with shaking at 250 rpm and then terminated by boiling in a water bath for 3 min. All reactions were carried out in triplicate.

### CRISPR-Cas9 Mediated Genome Editing

Electroporation of competent *E. coli* BL21(DE3) carrying pEcCas was carried out as described previously (Li et al., 2021). For λ-Red induction, L-arabinose (30 mM final concentration) was added to the culture. For genome editing, electroporation was performed using MicroPulser (Bio-Rad) with the default *E. coli* program (2 mm, 1.8 kV, and 6.1 ms). Each electroporation reaction included donor DNA and the plasmid pEcgRNA-Δ*sucA.* The electroporation mixture was suspended in 1 mL of LB medium right away. Cells were recovered by incubating for 2 h at 37°C before spreading on LB plates containing kanamycin (50 μg/mL) and spectinomycin (40 μg/mL) and incubating overnight at 37°C. Colonies were chosen at random and successful transformation was confirmed using colony PCR and DNA sequencing.

**FIGURE 1:**
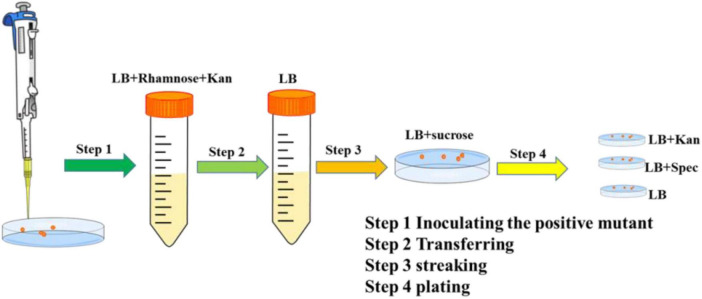
Elimination of the pEcgRNA and pEcCas plasmids.

A colony of the edited clone containing both pEcCas and pEcgRNA-Δ*sucA* was inoculated into 2 mL LB medium containing rhamnose (20 mM) and kanamycin (50 μg/ml) to eliminate the pEcgRNA-Δ*sucA* and pEcCas series plasmids (Figure 1). The culture was shaken at 220 rpm for 8 h before being transferred into a liquid LB medium without antibiotics and then grown for another 8 h. The cells were spread on LB plates containing 10 μg/mL sucrose and incubated overnight at 37°C on LB plates with or without kanamycin (50 μg/mL). LB plates, LB plates with kanamycin, and LB plates with spectinomycin were used to screen for positive colonies. The pEcgRNA-Δ*sucA*-cured colonies were used in the next round of *aceA* gene editing. The methods used for *aceA* gene editing were identical to those used for *sucA* deletion.

### Bioconversion Conditions

For the biotransformation, cells were suspended in the reaction mixture containing 0.5% (wt/v) glucose, 50 mM L-lysine, 10 mM Fe^2+^, 10 mM VC, 0.1%(w/v) Triton X-100 and cells with a final OD_600_ of 30 in PBS buffer (50 mM). The bioconversion reactions were performed at 30°C and 250 rpm on a shaker. Aliquots were taken at various time points, centrifuged, and heated at 100°C for 5 min to terminate the reaction. At least three independent experiments were performed for each strain.

### Analytical Methods

Cell growth was evaluated by measuring the absorbance at 600 nm using an UV spectrophotometer. For Fmoc derivatization, 50 μL of the culture supernatant was diluted with 250 μL of 100 mM borate buffer (pH 9.0). Diluted samples were derivatized with 10 mM Fmoc-Cl (TCI, Tokyo, Japan) in acetone (300 μL) and vigorously mixed and then reacted for 30 min. These mixtures were quenched with 600 μL of 25% acetonitrile/250 mM borate buffer (pH 5.5).

The amounts of L-lysine, L-Pip, and cis-3-HyPip were determined using a high-performance liquid chromatography system (Agilent 1260, Agilent Technologies, Santa Clara, CA, United States) with an HC-C18 column (Agilent Technologies, Santa Clara, CA, United States, 4.6 × 250 mm, 5 μm) and a flow rate of 1.0 mL/min at 40°C. The solvent gradient used was as follows: Buffer A [0.1% trifluoroacetic acid (TFA) water], B (0.1% TFA acetonitrile); 0–5 min, 30% B; 8–15 min, 40% B; 15–20 min, 40–50% B; 20–25 min, 50% B; 25–30 min, 50–90% B; 30–38 min, 90% B; 38–41 min, 90–30% B; and 41–45 min, 30% B.

## Results and Discussion

### Fine-Tuning of Cis-3-Hydroxypipecolic Acid Synthesis *via* the Optimization of the Gene Expression Strength

In our previous study, a dual-enzyme system containing SpLCD and GetF was constructed and reaction parameters were systematically optimized to biosynthesize cis-3-HyPip from L-lysine (Hu et al., 2022). However, the development of this whole-cell cascade catalyst is challenged by difficulty in balancing the rates of the two main reaction steps, namely, eliminating intermediate pipecolic acid accumulation and shifting the equilibrium toward cis-3-HyPip production. Tuning translation rates by varying the strength of RBS sequences has been widely used as an effective approach to balance the expression of biosynthetic enzymes in metabolic pathways and optimize the synthesis of target metabolites due to convenience and high efficiency (Liu et al., 2019b; Li et al., 2020).

**FIGURE 2:**
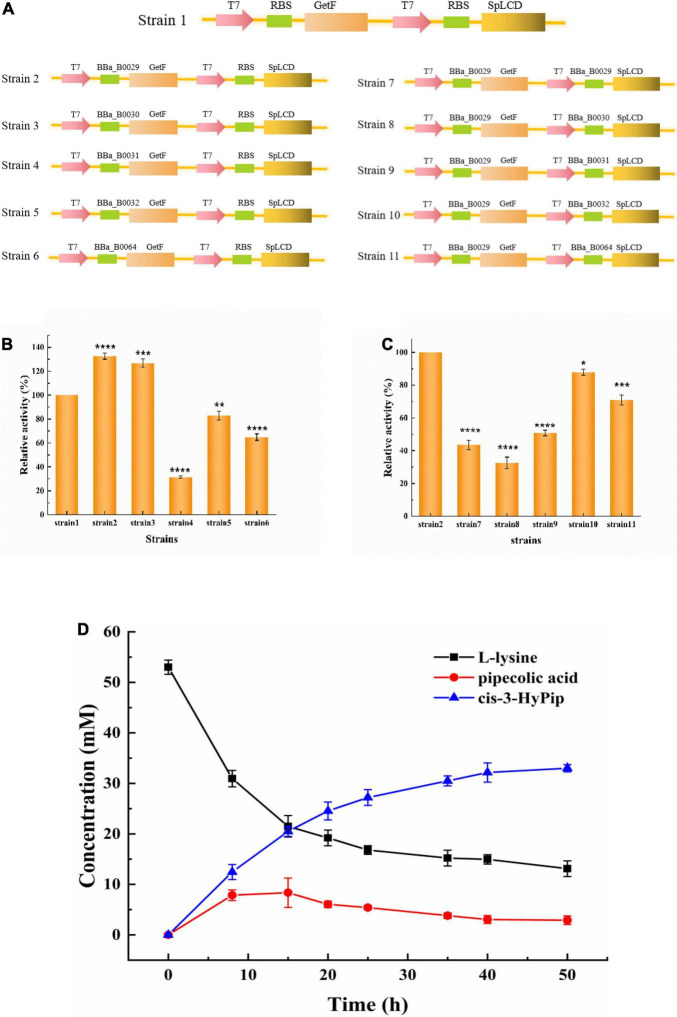
Whole-cell catalysis effects of strains with different RBS strengths. **(A)** Construction of different strains. **(B)** The effect of GetF RBS translation strength on catalysis. **(C)** The effect of SpLCD RBS translation strength on catalysis. **(D)** Time course of the bioconversion by the strain 2 (containing pETduet-1-29-*getf*-RBS-*splcd*). *P < 0.05, **P < 0.01, ***P < 0.001, ****P < 0.0001.

In the present study, the translation initiation rates of GetF and SpLCD were refined to enhance the titer of cis-3-HyPip. The expression levels of GetF and SpLCD were fine-tuned using RBS sequences with different translation initiation rates, and six RBS sequences were preliminarily selected in this study. T7 RBS originally existed in the Duet vectors, and five were from the iGEM Standard Parts and the RBS strength order was RBS 29 > 30 > RBS(T7) > 32 > 64 > 31 (Zhu et al., 2021). Using strain 1, which harbors pETduet-1-RBS(T7)-*getf*-RBS(T7)-*splcd*, as a starting strain, the GetF expression was combined with RBS sequences of various strengths to construct five strains (Figure 2A). As shown in Figure 2B, the efficiency of whole-cell catalysis was enhanced by increasing the strength of the RBS sequence. Finally, the highest activity was exhibited by the cells containing RBS 29 and the cis-3-HyPip productivity increased 30% through RBS optimization. Based on these results, the strain 2 harboring pETduet-1-29-*getf*-RBS(T7)-*splcd* as a starting strain, the SpLCD expression were combined with RBS sequences of various strengths to construct other five strains. As shown in Figure 2C, we found that the titers were much more impacted by the type of RBS used for GetF expression than that used for SpLCD expressions. Furthermore, it also showed that a moderate translational initiation rate of SpLCD is more suitable than the high or low rates, and that neither higher nor lower initiation rates not improved whole-cell activity. Similar results were also reported in previous studies, high-intensity RBS regulates the expression of non-rate-limiting enzymes, which may not result in a high yield of target products. Instead, medium-intensity RBS was observed to achieve a relatively highest yield (Alagesan et al., 2018; Sun et al., 2020). Finally, 32 mM cis-3-HyPip was obtained using strain 2 as the biocatalyst (Figure 2D). These results indicated that regulating gene expression by optimizing RBS strength could be utilized to solve the effect of rate-limiting steps and was useful for the target product yield improvement (Ge et al., 2022).

### Biocatalytic Response Upon Tricarboxylic Acid Cycle Redesign

GetF catalyzes the conversion of L-Pip to cis-3-HyPip, which requires 2-OG as a co-substrate, resulting in a high cost. Several studies have linked glutamate to the 2-OG conversion reaction (Wu et al., 2018; Sun et al., 2019). This reaction also produces H_2_O_2_, which can impair enzymatic activity (Niu et al., 2014; Jing et al., 2020). In the TCA cycle, 2-OG is an important intermediate metabolite. To simplify the technological processes and lower the overall production costs, a whole-cell biocatalyst was reconstructed by using a metabolic engineering strategy in *E. coli.* This intervention could introduce a 2-OG accumulation system, eliminating the need for additional 2-OG supplementation (Figure 4A).

**FIGURE 3:**
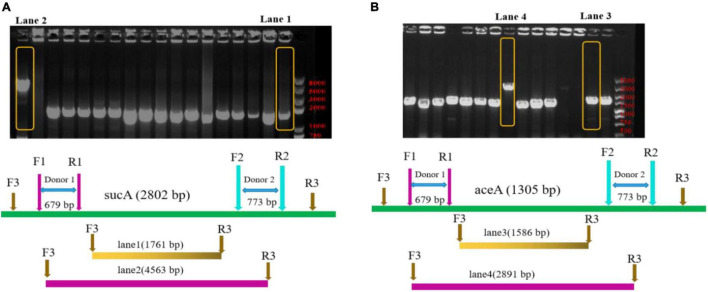
Polymerase chain reaction analysis of mutant strains. **(A)** Primers (F3/R3) were designed to target both ends of the *sucA* for the PCR detection of positive strains. The 1761-bp PCR-amplified fragment indicates that the *sucA* was knocked out, while the presence of a 4,563-bp fragment indicates that this gene was not knocked out. Lane 1: *E. coli BL21(DE3)* Δ*sucA*; lane 2: *E. coli* BL21(DE3). **(B)** Primers (F3/R3) were designed to target both ends of the *aceA* for the PCR detection of positive strains. The 1586-bp PCR-amplified fragment indicates that the *aceA* was knocked out, whereas the presence of a 2,891-bp fragment indicates that this gene was not knocked out. Lane 3: *E. coli* BL21(DE3) Δ*aceA*; Lane 4: *E. coli* BL21(DE3).

To engineer the metabolic flux of the TCA cycle, genes encoding *sucA* and *aceA* were separately knocked out in *E. coli* BL21(DE3). In this study, the *sucA* and *aceA* genes were knocked out in *BL21*(DE3) using the pEcCas/pEcgRNA system (Li et al., 2021). As shown in Figure 3A, the PCR amplification fragment was 4,563-bp (lane 2), indicated that *sucA* was not knocked out, while the PCR amplification fragment was 1,761-bp (lane 1), indicating that the *sucA* was knocked out. The PCR amplification fragment was 1,586-bp (lane 3), indicating that that it had been knocked out. By contrast, the 2,891-bp PCR amplification fragment (lane 4) indicated that *aceA* had not been knocked out (Figure 3B).

**FIGURE 4:**
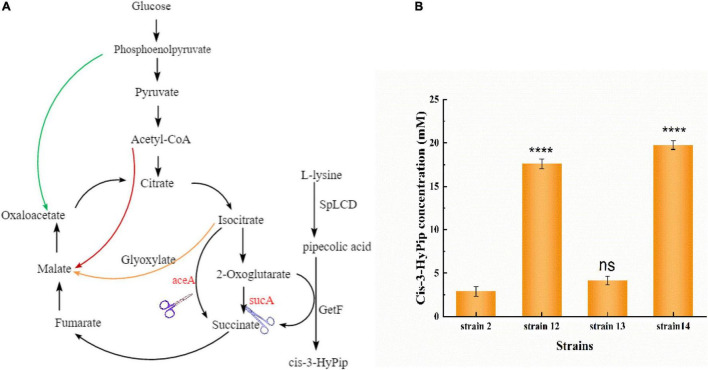
Reconstitution of the TCA cycle using GetF catalyzed reaction. **(A)** The manipulations to disrupt the TCA cycle and glyoxylate bypass. **(B)** Effect of *sucA* and *aceA* gene deletion on cis-3-HyPip production, strain 12 [*E. coli* BL21(DE3) Δ*sucA* pETduet-1-29-*getf*-RBS(T7)-*splcd*], strain 13 [*E. coli* BL21(DE3) Δ*aceA* pETduet-1-29-*getf*-RBS(T7)-*splcd*], strain 14 [*E. coli* BL21(DE3) Δ*sucA*Δ*aceA* pETduet-1-29-*getf*-RBS(T7)-*splcd*]. ****P < 0.0001.

Three *E. coli* mutants were obtained through knockout, and the plasmid pETduet-029-*getf*-RBS-*splcd* was transformed into these engineered strains, respectively. In the resulting recombinant strains (strain 12, strain 13, and strain 14) showed higher production of cis-3-HyPip than the starting strain 2. As shown in Figure 4B, the production of cis-3-HyPip by strain 2, and strain 12(Δ*sucA*), strain 13(Δ*aceA*), and strain 14(Δ*sucA*Δ*aceA*) was 3.1, 18, 4.4, and 20.1 mM. The strain 12(Δ*sucA)* showed the more obvious effect of increasing the yield of cis-3-HyPip. The deletion of *aceA*, the gene encoding the key enzyme for glyoxylate bypass, also caused a moderate increase in cis-3-HyPip yield, suggesting that *aceA* only played a minor role in 2-OG catabolism and only *sucA* mutation can completely block the conversion of 2-OG to succinate. These results indicate that using the redesigned TCA pathway to produce cis-3-HyPip in *E. coli* was effective. The TCA cycle can be engineered to adopt an alternative route without having significant impact on cell physiology (Lin et al., 2015).

### Over-Accumulation of a-Ketoglutarate by Enhancing Tricarboxylic Acid Flux

**FIGURE 5:**
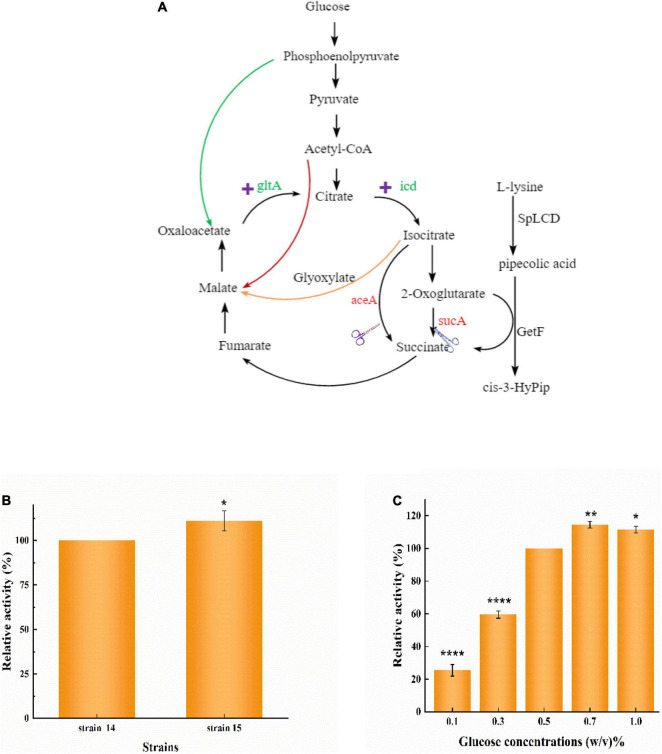
Increase whole-cell activity for cis-3-HyPip production by over-accumulation of 2-OG. **(A)** The combined manipulations to convert *E. coli* into an efficient whole-cell biocatalyst for cis-3-HyPip production. **(B)** Effect of overexpressing *glta* and *icd* on whole-cell catalysis. Strain 15 [*E. coli* BL21(DE3) △*sucA* △*aceA* pETduet-1-29-*getf*-RBS(T7)-*splcd* &pCDF-*glta*-*icd*], Strain 14 [*E. coli* BL21(DE3) △*sucA* △*aceA* pETduet-1-29-*getf*-RBS(T7)-*splcd*]. **(C)** Effects of glucose concentrations on whole-cell (strain 15) catalysis. *P < 0.05, **P < 0.01, ****P < 0.0001.

As previously reported, increasing the expression of genes encoding citrate synthase (*gltA*) and isocitrate dehydrogenase (*icd*) can increase the carbon flux to 2-OG (Zhang et al., 2018; Sun et al., 2019). Here, we used pCDFDuet-1, which contains the genes for citrate synthase (*gltA*) and isocitrate dehydrogenase (*icd*), to strengthen the TCA cycle (Figure 5A). Strain 15 [*E. coli* BL21(DE3) △*sucA* △*aceA* pETduet-1-29-*getf*-RBS(T7)-*splcd* &pCDF-*glta*-*icd*] was constructed on the basis of strain 14, and its produced slightly more than strain 14 (Figure 5B), indicating that overexpression of *gltA* and *icd* strengthen TCA cycle activity, thereby increasing the yield of cis-3-HyPip.

When glucose is taken up by *E. coli*, it is first broken down into pyruvate *via* glycolysis. Pyruvate is further converted to acetyl-CoA, which then enters the TCA cycle (Figure 5A). To increase the flux of the TCA cycle, different concentrations of glucose were also optimized. Our results showed that different glucose concentrations had a significant impact on cis-3-HyPip production in strain 15. As the glucose concentration increased from 0.1 to 0.7%, the production of cis-3-HyPip increased in concert (Figure 5C). At glucose concentrations higher than 0.7% (such as 0.7–1% w/v), no significant increase of cis-3-HyPip production was observed (Figure 5C). Such effects of glucose on cis-3-HyPip production were not observed in strain 2, which the TCA cycle was not coupled with the GetF catalyzed reaction. These results further demonstrated that the flux of the TCA cycle can be coupled with the catalyzed reaction (Ruan et al., 2019; An et al., 2020), and that the efficiency of the whole-cell biocatalysis for production can be improved by pushing the TCA cycle (Lin et al., 2015; Sun et al., 2019).

### Time Course of Cis-3-Hydroxypipecolic Acid Production

**FIGURE 6:**
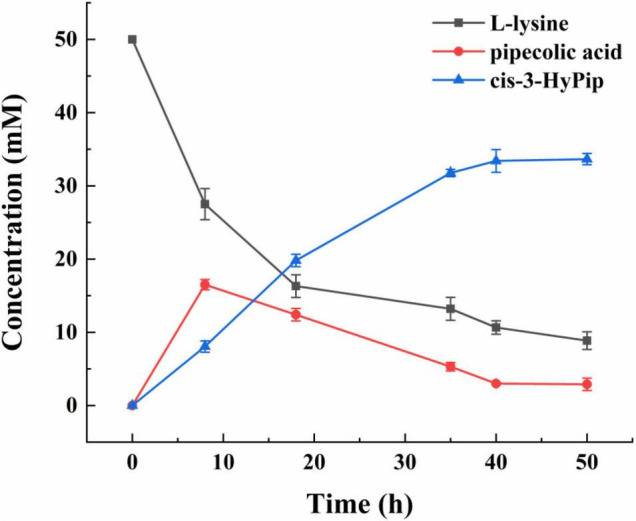
The time course of the bioconversion by strain 15 [*E. coli* BL21(DE3) △*sucA* △*aceA* pETduet-1-29-*getf*-RBS(T7)-*splcd* &pCDF-*glta*-*icd*].

Under the optimized conditions described above, a curve for whole-cell biocatalyst mediated conversion of L-lysine into cis-3-HyPip was obtained. As shown in Figure 6, at 0–20 h, the yield of cis-3-HyPip accumulated rapidly up to 21 mM. Subsequently, the rate of product generation increased slightly and slowly after 20 h. The product yield stabilized during the final phase (>40 h), which may be due to the decrease of thermal stability of GetF was the reason for the loss of catalytic activity. Finally, the reaction reached equilibrium when 33 mM cis-3-HyPip was produced after bioconversion for 50 h. Compared to that in previous studies (Table 1), the engineered strain used in this study displays the highest yield reported to date *via* whole-cell biotransformation, and without the addition of 2-OG.

**TABLE 1:** Currently used biological methods for the production of cis-3-HyPip.

Enzyme	Conversion process	Substrate	cis-3-HyPip production (mM)	References
cis-P3H	Purified enzyme	Pip 2-OG	0.17	Klein and Huettel, 2011
cis-P4H	Purified enzyme	L-Pip 2-OG	0.33	Klein and Huettel, 2011
SmP4H	Fermentation	L-Pip 2-OG	6.16	Koketsu et al., 2015
GetF	Purified enzyme	L-Pip 2-OG	7.8	Mattay and Huettel, 2017
GetF	Crude enzyme	L-Pip 2-OG	20	Zwick et al., 2021
SpLCD + GetF	Whole cell	2-OG L-lysine	25	Hu et al., 2022
SpLCD + GetF	Whole cell	L-lysine Glucose	33	This study

## Conclusion

In this study, we demonstrated synthetic biological strategies for enhancing cis-3-HyPip biosynthesis in *E. coli*. The translation rate was modulated by changing the strength of the RBS sequence to balance GetF and SpLCD expression and improve the titer of cis-3-HyPip. We also reconstituted the TCA cycle by knocking out the *sucA* and *aceA* genes, which reduced the addition of exogenous 2-OG and lowered the catalytic cost. The supply and catalytic efficiency of continuous 2-OG supplementation powered by glucose were improved by improving metabolic flux (gltA and icd) and optimizing glucose concentration. The maximum cis-3-HyPip titer was 33 mM. This is the first report of microbial biosynthesis of cis-3-HyPip that did not require the addition of 2-OG. Moreover, this strategy can also be applied to enzymes that catalyze 2-OG-coupled reactions and synthesize other functional compounds. It should be noted that there is still be much room for the improved production of cis-3-HyPip, for example, the directed evolution of GetF or high-throughput screening of GetF mutations can be studied in the future.

## Data Availability Statement

The original contributions presented in this study are included in the article/Supplementary Material, further inquiries can be directed to the corresponding author.

## Author Contributions

SH: methodology, data curation, writing – original draft, and investigation. YL: methodology. HL: supervision. KC: conceptualization, supervision, project administration, and funding acquisition. AZ: review and editing. PO: project administration and funding acquisition. All authors contributed to the article and approved the submitted version.

## Conflict of Interest

The authors declare that the research was conducted in the absence of any commercial or financial relationships that could be construed as a potential conflict of interest.

## Publisher’s Note

All claims expressed in this article are solely those of the authors and do not necessarily represent those of their affiliated organizations, or those of the publisher, the editors and the reviewers. Any product that may be evaluated in this article, or claim that may be made by its manufacturer, is not guaranteed or endorsed by the publisher.

## Footnotes

1
http://parts.igem.org/Ribosome_Binding_Sites/Catalog

## Abbreviations

cis-3-HyPip: cis-3-hydroxypipecolic acid
2-OG: α -ketoglutarate
HyPips: hydroxypipecolic acids
L-Pip: L-pipecolic acid
IPTG: isopropyl β-D -1-thiogalactopyranoside
PCR: polymerase chain reaction
RBS: ribosomal binding site
TCA: tricarboxylic acid cycle
VC: L-ascorbate .
