# Supplementary material for: Designing of an Efficient Whole-Cell Biocatalyst System for Converting L-Lysine Into Cis-3-Hydroxypipecolic Acid
Source: Front Microbiol. 2022 Jun 27;13:945184. doi: 10.3389/fmicb.2022.945184 (PMC9271919; doi:10.3389/fmicb.2022.945184)
Supplement: Supplementary file 1 [file Table_1.DOCX]

**Designing of an efficient whole-cell biocatalyst** **system for converting L-lysine into cis-3-hydroxypipecolic acid**

Shewei Hu, Yangyang Li, Alei Zhang, Hui Li, Kequan Chen*, Pingkai Ouyang

*State Key Laboratory of Materials-Oriented Chemical Engineering, College of Biotechnology and Pharmaceutical Engineering, Nanjing Tech University, Nanjing, 211800, P.R. China*

*Corresponding author: Tel.: +86-138-1418-0652

E-mail address: kqchen@njtech.edu.cn

Table S1 *E. coli* strains and plasmids used in this study

| strains or plasmids | Relevant characteristics | Source |
| --- | --- | --- |
| Strains |  |  |
| *E. coli* DH5α | Cloning host | Novagen |
| *E. coli* BL21(DE3) | Protein expression host | Novagen |
| *E. coli* DB3.1 | Cloning host for pEcgRNA | Shanghai Institutes for Biological Sciences |
| Strain1 | *E. coli* BL21(DE3) harboring plasmid 1 | This lab |
| Strain2  Strain3  Strain4  Strain5  Strain6  Strain7  Strain8  Strain9  Strain10  Strain11  Strain12  Strain13  Strain14  Strain15 | *E. coli* BL21(DE3) harboring plasmid 2  *E. coli* BL21(DE3) harboring plasmid 3  *E. coli* BL21(DE3) harboring plasmid 4  *E. coli* BL21(DE3) harboring plasmid 5  *E. coli* BL21(DE3) harboring plasmid 6  *E. coli* BL21(DE3) harboring plasmid 7  *E. coli* BL21(DE3) harboring plasmid 8  *E. coli* BL21(DE3) harboring plasmid 9  *E. coli* BL21(DE3) harboring plasmid 10  *E. coli* BL21(DE3) harboring plasmid 11  *E. coli* BL21(DE3) Δ*sucA*  harboring plasmid 2  *E. coli* BL21(DE3) Δ*aceA*  harboring plasmid 2  *E. coli* BL21(DE3) Δ*sucA*Δ*aceA* harboring plasmid 2  *E. coli* BL21(DE3) Δ*suc*AΔ*aceA* harboring plasmid 2 and plasmid 12 | This study  This study  This study  This study  This study  This study  This study  This study  This study  This study  This study  This study  This study  This study |
| Plasmids  pCDFDuet-1 | double T7 promoters, CDF 13 ori, Sm^R^ | Novagen |
| pETDuet-1 | double T7 promoters, pBR322 ori, Amp^R^ | Novagen |
| Plasmid1  Plasmid2  Plasmid3  Plasmid4  Plasmid5  Plasmid6  Plasmid7  Plasmid8  Plasmid9  Plasmid10  Plasmid11  Plasmid12  Plasmid13  Plasmid14  Plasmid15  Plasmid16  Plasmid17  Plasmid18 | pETDuet-1 with RBS T7 linked with GetF and RBS T7 with SpLCD  pETDuet-1 with RBS 029 linked with GetF and RBS T7 with SpLCD  pETDuet-1 with RBS 030 linked with GetF and RBS T7 with SpLCD  pETDuet-1 with RBS 31 linked with GetF and RBS T7 with SpLCD  pETDuet-1 with RBS 032 linked with GetF and RBS T7 with SpLCD  pETDuet-1 with RBS 064 linked with GetF and RBS T7 with SpLCD  pETDuet-1 with RBS 029 linked with GetF and RBS 029 with SpLCD  pETDuet-1 with RBS 029 linked with GetF and RBS 030 with SpLCD  pETDuet-1 with RBS 029 linked with GetF and RBS 031 with SpLCD  pETDuet-1 with RBS 029 linked with GetF and RBS 32 with SpLCD  pETDuet-1 with RBS 029 linked with GetF and RBS 064 with SpLCD  pCDFDuet-1with glta and icd  pEcCas  pEcgRNA  pEcgRNA -*aceA*-N20A  pEcgRNA- *aceA*-N20B  pEcgRNA -*sucA*-N20A  pEcgRNA -*sucA*-N20B | This lab  This study  This study  This study  This study  This study  This study  This study  This study  This study  This study  This study  Shanghai Institutes for Biological Sciences  Shanghai Institutes for Biological Sciences  This study  This study  This study  This study |

Table S2 Primers for construction of various RBS site used in this study

| Plasmid2F ACTTTAAGTTCACACAGGAAACCATATACCATGGGCAGCAGCCATCACCATC  Plasmid2R  GGTATATGGTTTCCTGTGTGAACTTAAAGTTAAACAAAATTATTTCTAGAGGG |
| --- |

Plasmid3F

ACTTTAAGATTAAAGAGGAGAAAATATACCATGGGCAGCAGCCATCACCATC

Plasmid3R

GGTATATTTTCTCCTCTTTAATCTTAAAGTTAAACAAAATTATTTCTAGAGGG

Plasmid4F

ACTTTAAG TCACACAGGAAACC ATATACCATGGGCAGCAGCCATCACCATC

Plasmid4R

GGTATAT TTTCTCCTCTTTAATCTTAAAGTTAAACAAAATTATTTCTAGAGGG

Plasmid5F

ACTTTAAG TCACACAGGAAAG ATATACCATGGGCAGCAGCCATCACCATC

Plasmid5R

GGTATAT CTTTCCTGTGTGACTTAAAGTTAAACAAAATTATTTCTAGAGGG

Plasmid6F

ACTTTAAG AAAGAGGGGAAAATATACCATGGGCAGCAGCCATCACCATC

Plasmid6R

GGTATAT TTTCCCCTCTTTCTTAAAGTTAAACAAAATTATTTCTAGAGGG

Plasmid7F

AGTATAAGTTCACACAGGAAACCATATACATATGGCAGAT

Plasmid7R

CATATGTATATGGTTTCCTGTGTGAACTTATACTTAACTAATATACTAA

Plasmid8F

AGTATAAGATTAAAGAGGAGAAAATATACATATGGCAGAT

Plasmid8R

CATATGTATATTTTCTCCTCTTTAATCTTATACTTAACTAATATACTAA

Plasmid9F

AGTATAAG TCACACAGGAAACC ATATACATATGGCAGAT

Plasmid9R

CATATGTATATTTTCTCCTCTTTAATCTTATACTTAACTAATATACTAA

Plasmid10F

AGTATAAG TCACACAGGAAAG ATATACATATGGCAGAT

Plasmid10R CATATGTATATCTTTCCTGTGTGACTTATACTTAACTAATATACTAA

Plasmid11F AGTATAAGAAAGAGGGGAAAATATACATATGGCAGAT

Plasmid11R CATATGTATATTTTCCCCTCTTTCTTATACTTAACTAATATACTAA

|  |
| --- |

Table S3 Primers for gene editing used in this study. DNA fragment (5′–3′)

| *aceA* F1 CCTGCCATAAACGCGGTGC |
| --- |

*aceA* R1 TTGTTGTTGCGTGCAGATGCTCCATAGTTATGTG

*aceA* F2 GCATCTGCACGCAACAACAACCGTTGCTG

*aceA*R2 CGCCACCTGTAAATGACTTTCC

*aceA*F3 ATCGCGTCCTGCCAGACAGAC

*aceA*R3 GGTGTGATCAGTTTGCCTACCAGC

*aceA*-N20A-Fa TAGTCGTCTGGCAGCTGACGTGAC

*aceA*-N20A-Ra AAACGTCACGTCAGCTGCCAGACG

*aceA*-N20B-Fb TAGTGCTTCCGCATCGGCAACGAT

*aceA*-N20B-Rb AAACATCGTTGCCGATGCGGAAGC

*sucA*F1 AGAAGATTGTGATTCGCCCGC

*sucA*R1 TTGTGTATCCTTTATCGTGATCCCTTAAGCATCTTTTT

*sucA*F2 CTTAAGGGATCACGATAAAGGATACACAATGAGTAGCGTAGA

*sucA*R2 GCTTCAACCACCGCTTTCACG

*sucA*F3 GATGAAGGTCGCGACATGATGCT

*sucA*R3 TGCTGACGTCGAAATAGTTGTGG

*sucA*-N20A-Fa TAGTTTACCGATATCAGTACAGTA

*sucA*-N20A-Ra AAACTACTGTACTGATATCGGTAA

*sucA*-N20B-Fb TAGT GCGATTGCGTAGTGGCAGAG

*sucA*-N20B-Rb AAAC CTCTGCCACTACGCAATCGC

|  |
| --- |

Table S4 Primers for construction plasmid pCDFduet-*glta*-*icd* used in this study. DNA fragment (5′–3′)

| glta(*EcoR I*) CGGAATTCatggctgatacaaaagcaaaactc  glta(*Hind III*) cccaagcttttaacgcttgatatcgcttttaaagtc  icd(*Bgl II*) gaagatctatggaaagtaaagtagttgttccg |
| --- |

icd(*Xho I*) ccgctcgagttacatgttttcgatgatcgcgt

|  |
| --- |

Table S5 Donor DNA sequence Homologous DNA fragment (5′–3′)

| *aceA* |
| --- |

CCTGCCATAAACGCGGTGCTTTTGCGATGGGCGGCATGGCGGCGTTTATTCCGAGCAAAGATGAAGAGCACAATAACCAGGTGCTCAACAAAGTAAAAGCGGATAAATCGCTGGAAGCCAATAACGGTCACGATGGCACATGGATCGCTCACCCAGGCCTTGCGGACACGGCAATGGCGGTATTCAACGACATTCTCGGCTCCCGTAAAAATCAGCTTGAAGTGATGCGCGAACAAGACGCGCCGATTACTGCCGATCAGCTGCTGGCACCTTGTGATGGTGAACGCACCGAAGAAGGTATGCGCGCCAACATTCGCGTGGCTGTGCAGTACATCGAAGCGTGGATCTCTGGCAACGGCTGTGTGCCGATTTATGGCCTGATGGAAGATGCGGCGACGGCTGAAATTTCCCGTACCTCGATCTGGCAGTGGATCCATCATCAAAAAACGTTGAGCAATGGCAAACCGGTGACCAAAGCCTTGTTCCGCCAGATGCTGGGCGAAGAGATGAAAGTCATTGCCAGCGAACTGGGCGAAGAACGTTTCTCCCAGGGGCGTTTTGACGATGCCGCACGCTTGATGGAACAGATCACCACTTCCGATGAGTTAATTGATTTCCTGACCCTGCCAGGCTACCGCCTGTTAGCGTAAACCACCACATAACTATGGAGCATCTGCACGCAACAACAACCGTTGCTGACTGTAGGCCGGATAAGGCGTTCACGCCGCATCCGGCAATCGGTGCACGATGCCTGATGCGACGCTTGCGCGTCTTATCATGCCTACAGCCGTTGCCGAACGTAGGCTGGATAAGGCGTTTACGCCGCATCCGGCAATTCTCTGCTCCTGATGAGGGCGCTAAATGCCGCGTGGCCTGGAATTATTGATTGCTCAAACCATTTTGCAAGGCTTCGATGCTCAGTATGGTCGATTCCTCGAAGTGACCTCCGGTGCGCAGCAGCGTTTCGAACAGGCCGACTGGCATGCTGTCCAGCAGGCGATGAAAAACCGTATCCATCTTTACGATCATCACGTTGGTCTGGTCGTGGAGCAACTGCGCTGCATTACTAACGGCCAAAGTACGGACGCGGCATTTTTACTACGTGTTAAAGAGCATTACACCCGGCTGTTGCCGGATTACCCGCGCTTCGAGATTGCGGAGAGCTTTTTTAACTCCGTGTACTGTCGGTTATTTGACCACCGCTCGCTTACTCCCGAGCGGCTTTTTATCTTTAGCTCTCAGCCAGAGCGCCGCTTTCGTACCATTCCCCGCCCGCTGGCGAAAGACTTTCACCCCGATCACGGCTGGGAATCTCTACTGATGCGCGTTATCAGCGACCTACCGCTGCGCCTGCGCTGGCAGAATAAAAGCCGTGACATCCATTACATTATTCGCCATCTGACGGAAACGCTGGGGACAGACAACCTCGCGGAAAGTCATTTACAGGTGGCG

*sucA*

AGAAGATTGTGATTCGCCCGCTGCCAGGTTTACCGGTGATCCGCGATTTGGTGGTAGACATGGGACAATTCTATGCGCAATATGAGAAAATTAAGCCTTACCTGTTGAATAATGGACAAAATCCGCCAGCTCGCGAGCATTTACAGATGCCAGAGCAGCGCGAAAAACTCGACGGGCTGTATGAATGTATTCTCTGCGCATGTTGTTCAACCTCTTGTCCGTCTTTCTGGTGGAATCCCGATAAGTTTATCGGCCCGGCAGGCTTGTTAGCGGCATATCGTTTCCTGATTGATAGCCGTGATACCGAGACTGACAGCCGCCTCGACGGTTTGAGTGATGCATTCAGCGTATTCCGCTGTCACAGCATCATGAACTGCGTCAGTGTATGTCCGAAGGGGCTGAACCCGACGCGCGCCATCGGCCATATCAAGTCGATGTTGTTGCAACGTAATGCGTAAACCGTAGGCCTGATAAGACGCGCAAGCGTCGCATCAGGCAACCAGTGCCGGATGCGGCGTGAACGCCTTATCCGGCCTACAAGTCATTACCCGTAGGCCTGATAAGCGCAGCGCATCAGGCGTAACAAAGAAATGCAGGAAATCTTTAAAAACTGCCCCTGACACTAAGACAGTTTTTAAAGGTTCCTTCGCGAGCCACTACGTAGACAAGAGCTCGCAAGTGAACCCCGGCACGCACATCACTGTGCGTGGTAGTATCCACGGCGAAGTAAGCATAAAAAAGATGCTTAAGGGATCACGATAAAGGATACACAATGAGTAGCGTAGATATTCTGGTCCCTGACCTGCCTGAATCCGTAGCCGATGCCACCGTCGCAACCTGGCATAAAAAACCCGGCGACGCAGTCGTACGTGATGAAGTGCTGGTAGAAATCGAAACTGACAAAGTGGTACTGGAAGTACCGGCATCAGCAGACGGCATTCTGGATGCGGTTCTGGAAGATGAAGGTACAACGGTAACGTCTCGTCAGATCCTTGGTCGCCTGCGTGAAGGCAACAGCGCCGGTAAAGAAACCAGCGCCAAATCTGAAGAGAAAGCGTCCACTCCGGCGCAACGCCAGCAGGCGTCTCTGGAAGAGCAAAACAACGATGCGTTAAGCCCGGCGATCCGTCGCCTGCTGGCTGAACACAATCTCGACGCCAGCGCCATTAAAGGCACCGGTGTGGGTGGTCGTCTGACTCGTGAAGATGTGGAAAAACATCTGGCGAAAGCCCCGGCGAAAGAGTCTGCTCCGGCAGCGGCTGCTCCGGCGGCGCAACCGGCTCTGGCTGCACGTAGTGAAAAACGTGTCCCGATGACTCGCCTGCGTAAGCGTGTGGCAGAGCGTCTGCTGGAAGCGAAAAACTCCACCGCCATGCTGACCACGTTCAACGAAGTCAACATGAAGCCGATTATGGATCTGCGTAAGCAGTACGGTGAAGCGTTTGAAAAACGCCACGGCATCCGTCTGGGCTTTATGTCCTTCTACGTGAAAGCGGTGGTTGAAGC

|  |
| --- |
|  |
|  |
|  |
| Table S6 icd and glta genes sequences and enzyme cleavage site |
| *glta* (*EcoR I*/*Hind III*) |

GAATTCatggctgatacaaaagcaaaactcaccctcaacggggatacagctgttgaactggatgtgctgaaaggcacgctgggtcaagatgttattgatatccgtactctcggttcaaaaggtgtgttcacctttgacccaggcttcacttcaaccgcatcctgcgaatctaaaattacttttattgatggtgatgaaggtattttgctgcaccgcggtttcccgatcgatcagctggcgaccgattctaactacctggaagtttgttacatcctgctgaatggtgaaaaaccgactcaggaacagtatgacgaatttaaaactacggtgacccgtcataccatgatccacgagcagattacccgtctgttccatgctttccgtcgcgactcgcatccaatggcagtcatgtgtggtattaccggcgcgctggcggcgttctatcacgactcgctggatgttaacaatcctcgtcaccgtgaaattgccgcgttccgcctgctgtcgaaaatgccgaccatggccgcgatgtgttacaagtattccattggtcagccatttgtttacccgcgcaacgatctctcctacgccggtaacttcctgaatatgatgttctccacgccgtgcgaaccgtatgaagttaatccgattctggaacgtgctatggaccgtattctgatcctgcacgctgaccatgaacagaacgcctctacctccaccgtgcgtaccgctggctcttcgggtgcgaacccgtttgcctgtatcgcagcaggtattgcttcactgtggggacctgcgcacggcggtgctaacgaagcggcgctgaaaatgctggaagaaatcagctccgttaaacacattccggaatttgttcgtcgtgcgaaagacaaaaatgattctttccgcctgatgggcttcggtcaccgcgtgtacaaaaattacgacccgcgcgccaccgtaatgcgtgaaacctgccatgaagtgctgaaagagctgggcacgaaggatgacctgctggaagtggctatggagctggaaaacatcgcgctgaacgacccgtactttatcgagaagaaactgtacccgaacgtcgatttctactctggtatcatcctgaaagcgatgggtattccgtcttccatgttcaccgtcattttcgcaatggcacgtaccgttggctggatcgcccactggagcgaaatgcacagtgacggtatgaagattgcccgtccgcgtcagctgtatacaggatatgaaaaacgcgactttaaaagcgatatcaagcgttaaaagctt

*icd* (*Bgl II* / *XhoI*)

agatctatggaaagtaaagtagttgttccggcacaaggcaagaagatcaccctgcaaaacggcaaactcaacgttcctgaaaatccgattatcccttacattgaaggtgatggaatcggtgtagatgtaaccccagccatgctgaaagtggtcgacgctgcagtcgagaaagcctataaaggcgagcgtaaaatctcctggatggaaatttacaccggtgaaaaatccacacaggtttatggtcaggacgtctggctgcctgctgaaactcttgatctgattcgtgaatatcgcgttgccattaaaggtccgctgaccactccggttggtggcggtattcgctctctgaacgttgccctgcgccaggaactggatctctacatctgcctgcgtccggtacgttactatcagggcactccaagcccggttaaacaccctgaactgaccgatatggttatcttccgtgaaaactcggaagacatttatgcgggtatcgaatggaaagcagactctgccgacgccgagaaagtgattaaattcctgcgtgaagagatgggggtgaagaaaattcgcttcccggaacattgtggtatcggtattaagccgtgttcggaagaaggcaccaaacgtctggttcgtgcagcgatcgaatacgcaattgctaacgatcgtgactctgtgactctggtgcacaaaggcaacatcatgaagttcaccgaaggagcgtttaaagactggggctaccagctggcgcgtgaagagtttggcggtgaactgatcgacggtggcccgtggctgaaagttaaaaacccgaacactggcaaagagatcgtcattaaagacgtgattgctgatgcattcctgcaacagatcctgctgcgtccggctgaatatgatgttatcgcctgtatgaacctgaacggtgactacatttctgacgccctggcagcgcaggttggcggtatcggtatcgcccctggtgcaaacatcggtgacgaatgcgccctgtttgaagccacccacggtactgcgccgaaatatgccggtcaggacaaagtaaatcctggctctattattctctccgctgagatgatgctgcgccacatgggttggaccgaagcggctgacttaattgttaaaggtatggaaggcgcaatcaacgcgaaaaccgtaacctatgacttcgagcgtctgatggatggcgctaaactgctgaaatgttcagagtttggtgacgcgatcatcgaaaacatgtaactcgag

|  |
| --- |
